# Supplementary material for: Distributed sensing of earthquakes and ocean-solid Earth interactions on seafloor telecom cables
Source: Nat Commun. 2019 Dec 18;10:5777. doi: 10.1038/s41467-019-13793-z (PMC6920424; doi:10.1038/s41467-019-13793-z)
Supplement: Supplementary file 1 — Supplementary Information [file 41467_2019_13793_MOESM1_ESM.pdf]

Supplementary Information

Supplementary Note 1: Main characteristics of the MEUST-NUMerEnv cable

The MEUST-NUMerEnv cable straddles several oceanic domains of the north Mediterranean margin: a shallow continental shelf, a steep continental slope (12% average slope), and a 2500 m-deep oceanic plain. Its armoring is adapted for the specific conditions for each domain and the associated risks of damage: Double Armoring Heavy (DAH) for the first 2.1 km, Single Armoring Heavy (SAH) for the next 15.1 km, and Light-Weight Protection (LWP) for the remaining 24 km. On land, an extra 1.4 km of fiber connects the cable to the shore station. From that shore station to the seafloor termination point of the cable, the OF remains of the same type (Corning LEAF©).

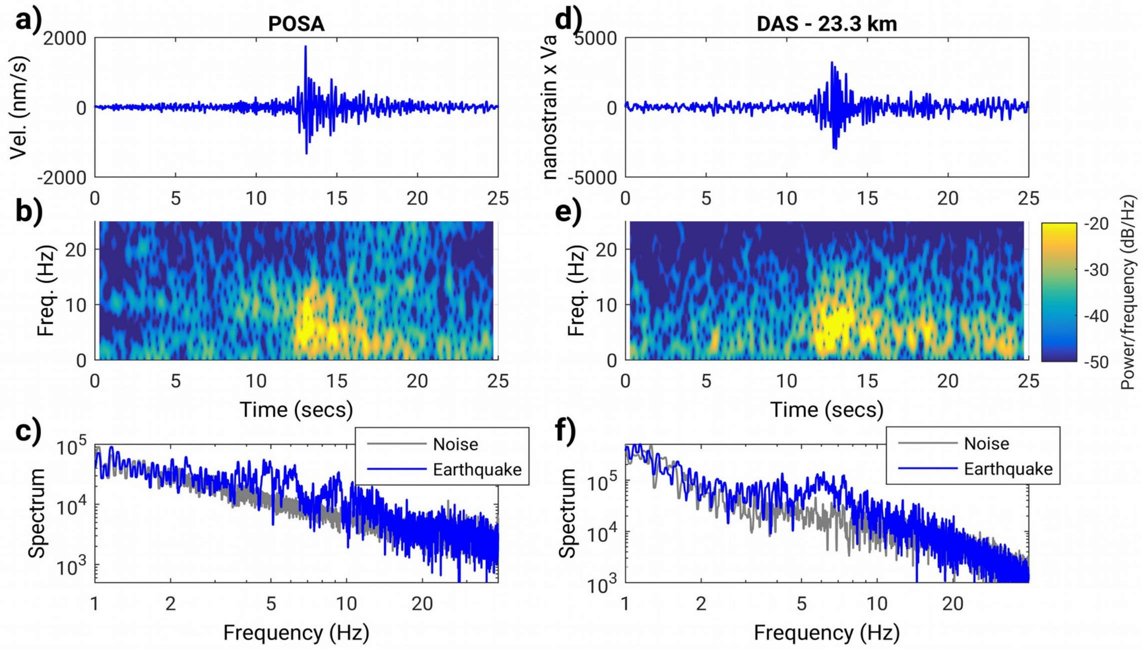

Supplementary Figure 1: Velocity records of M1.9 earthquake at the North component of station POSA compared to strain OF measurements. Comparison of a M1.9 earthquake recorded by the on-land station POSA (a, b,c) and by the OF cable at km 23.3 (d,e,f). a) and d) time signals are filtered between 2 and 20 Hz; b) and d) spectrograms of the signals filtered between 2 and 25 Hz; c) and f) spectrum of the earthquake (blue) and the noise (grey). The signals recorded on the optic fiber have been converted to strain through a time integration, and stacked with a normal moveout correction on a 384 m section. For comparison with the POSA velocity record, the signal was also multiplied by the apparent velocity (Va=2155 m/s), assuming that the record corresponds to an incoming plane wave.

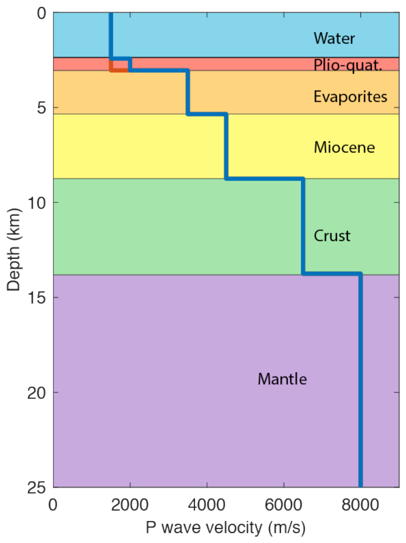

Supplementary Figure 2: P-wave seismic velocity model used to model the secondary microseismic noise. Model derived from (*23*)

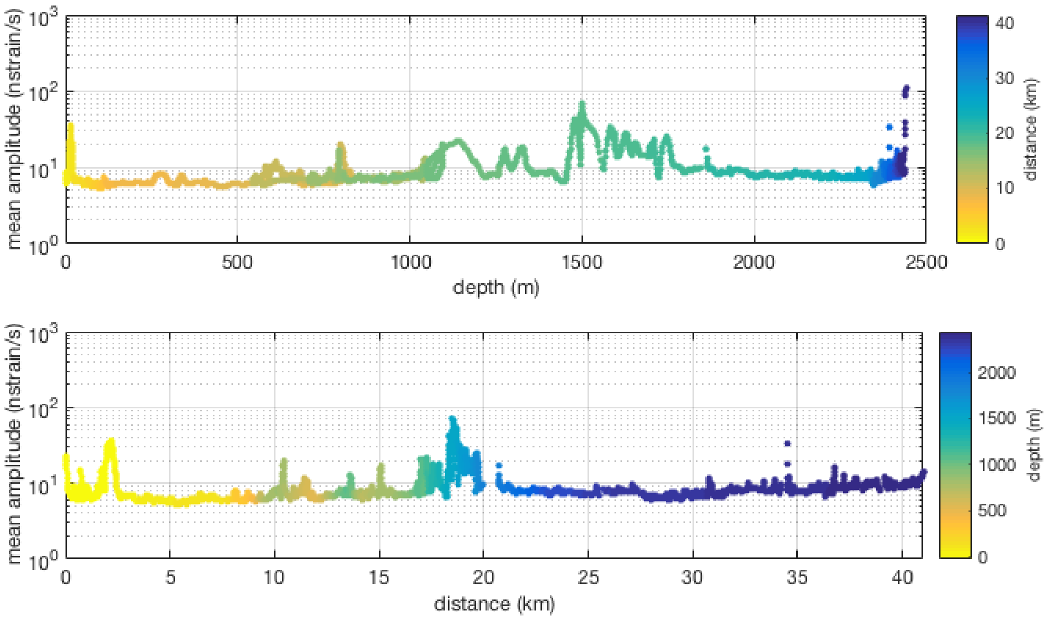

Supplementary Figure 3: Variations in the mean strain-rate during an earthquake. Mean nano strain-rate amplitude as a function of depth (top) and distance (bottom) recorded during the magnitude 1.9 earthquake. The earthquake signal is filtered between 2.5 and 15Hz.

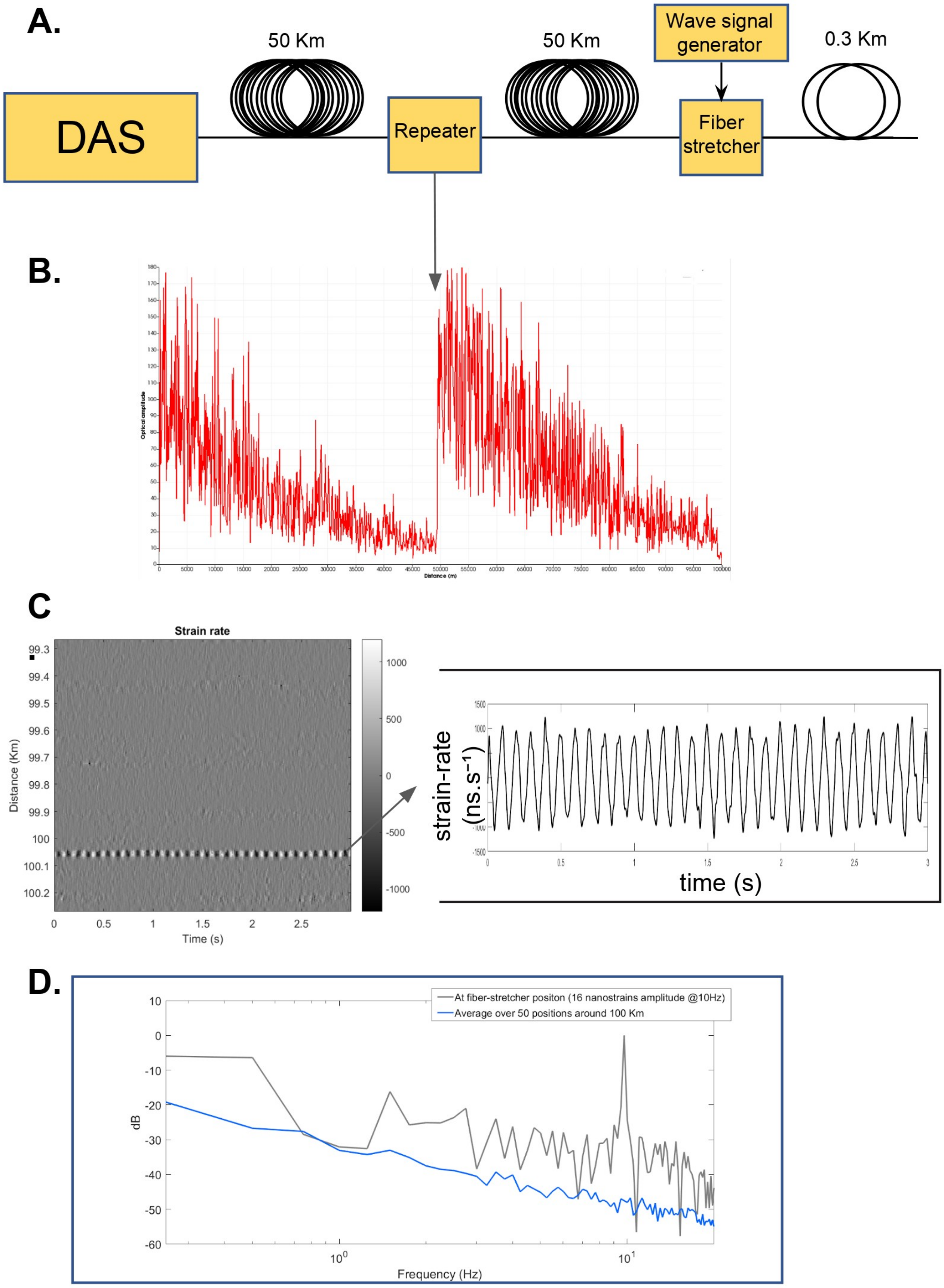

Supplementary Figure 4: Lab test of DAS measurement at 100km with a bi-directional optical amplifer. A) A standard DAS acquisition unit is connect to two 50 km fiber spools with a bi-directional optical amplifer in between to re-amplify the signal. At the end of the fiber, a fiber stretcher applies controlled 16 nanostrain vibrations at 10Hz. B) Optical amplitude decay of the optical signal along the fiber. C) The strain-rate signal measured by the DAS unit at the location of the fiber stretcher. D) Spectrogram of strain at the fiber-

stretcher position (grey) or averaged at 50 points outside that area (blue). These figures demonstrate the ability to extend the range of a standard DAS unit without compromising the quality of the measurement.
