# Supplementary material for: Distributed sensing of earthquakes and ocean-solid Earth interactions on seafloor telecom cables
Source: Nat Commun. 2019 Dec 18;10:5777. doi: 10.1038/s41467-019-13793-z (PMC6920424; doi:10.1038/s41467-019-13793-z)
Supplement: Supplementary file 3 — Description of Additional Supplementary Files [file 41467_2019_13793_MOESM3_ESM.pdf]

Description of Additional Supplementary Files

File name: Supplementary Movie 1

Description: Seismic signal recorded over the continental shelf. Perspective view of the bathymetry and the first 8km of seafloor cable. The movie presents one minute of evolution of the amplitude of the seismic signal. The amplitude fluctuations illustrate the motion of the vertical pressure exerted by ocean surface waves propagating at the surface. These wave propagate predominantly towards the shore with a wavelength controlled by the water depth.
